# Variation in nocturnality and circadian activity rhythms between photoresponsive F344 and nonphotoresponsive Sprague Dawley rats

**DOI:** 10.1186/1740-3391-6-8

**Published:** 2008-09-09

**Authors:** Cheryl D Seroka, Cynthia E Johnson, Paul D Heideman

**Affiliations:** 1 Department of Biology, College of William and Mary, Williamsburg, VA 23187, USA

## Abstract

**Background:**

Variation in circadian rhythms and nocturnality may, hypothetically, be related to or independent of genetic variation in photoperiodic mediation of seasonal changes in physiology and behavior. We hypothesized that strain variation in photoperiodism between photoperiodic F344 rats and nonphotoperiodic Harlan Sprague Dawley (HSD) rats might be caused by underlying variation in clock function. We predicted that HSD rats would have more activity during the day or subjective day, longer free-running rhythms, poor entrainment to short day length, and shorter duration of activity, traits that have been associated with nonphotoperiodism in other laboratory rodent species, relative to F344 rats. An alternative hypothesis, that differences are due to variation in melatonin secretion or responses to melatonin, predicts either no such differences or inconsistent combinations of differences.

**Methods:**

We tested these predictions by examining activity rhythms of young male F344 and HSD rats given access to running wheels in constant dark (DD), short day length (L8:D16; SD), and long day length (L16:D8; LD). We compared nocturnality (the proportion of activity during night or subjective night), duration of activity (alpha), activity onset and offset, phase angle of entrainment, and free running rhythms (tau) of F344 and HSD rats.

**Results:**

HSD rats had significantly greater activity during the day, were sometimes arrhythmic in DD, and had significantly longer tau than F344 rats, consistent with predictions. However, HSD rats had significantly longer alpha than F344 rats and both strains entrained to SD, inconsistent with predictions.

**Conclusion:**

The ability of HSD rats to entrain to SD, combined with longer alpha than F344 rats, suggests that the circadian system of HSD rats responds correctly to SD. These data offer best support for the alternative hypothesis, that differences in photoresponsiveness between F344 and HSD rats are caused by non-circadian differences in melatonin secretion or the response to melatonin.

## Background

Precise timing of both circadian and seasonal changes in physiology and behavior are important for animals [1]. Biologically appropriate daily and seasonal timing depends upon normal and precise function of the circadian system [2-4]. Genetic variation for circadian clock function can affect circadian physiological rhythms, daily cyclical behaviors, onset and offset of activity, melatonin rhythms, and regulation of seasonal physiological changes in energetics and reproduction [4-6]. Such genetic variation may affect animal and human health and function through effects on biological rhythms and related physiological systems [4,7-9]. Particularly important for the circadian system may be pleiotropic variation that could cause correlated variation in more than one trait, thereby affecting multiple body systems [e.g., examples of gene knockout studies reviewed in [4]].

Studies on laboratory colonies of hamsters have described pleiotropic genetic variation in the circadian clock that also causes variation in photoperiodism [10-14]. In Siberian hamsters, for example, genetically nonphotoperiodic individuals in short photoperiod have a delayed, lower amplitude nocturnal rise in pineal melatonin, a 4.5 hour delay in the onset of nightly activity, a longer free-running activity period (*tau*), a shorter duration of running wheel activity (*alpha*), and some nonphotoperiodic individuals are arrhythmic, relative to photoperiodic individuals [10,11]. In *tau *mutant Syrian hamsters, the free running rhythm is too short for proper entrainment to a short photoperiod, leading to an inability to produce a short photoperiod melatonin pattern [15]. In contrast, studies on natural populations of rodents have reported genetic variation in seasonal photoperiodic traits which have not been found to be related to circadian variation [16-20], suggesting independent sources of variation. It is not clear how commonly variation in photoperiodic seasonality is correlated with circadian rhythms, and additional models that relate genetic variation in circadian and seasonal function would be useful.

Strains of laboratory rats vary both in circadian rhythms [21-25] and in photoperiodic responses that include reproduction, food intake, and body mass [26-30]. The F344/NHsd strain of rats suppresses reproduction, food intake, and somatic growth in short photoperiods, while Harlan Sprague Dawley (HSD) rats do not. F344 rats are becoming a model for mechanisms of photoresponsiveness [31], and may be useful models for the study of correlated genetic variation in rhythms, regulation of appetite and body mass, and reproduction. A recent comparison of the rhythm of excretion of the major metabolite of melatonin, 6-sulfatoxymelatonin, between photoperiodic F344 rats and nonphotoperiodic HSD rats suggests that both strains have long duration melatonin secretion in short photoperiod and short duration melatonin secretion in long photoperiod [32]. Thus, both photoresponsive F344 and nonphotoresponsive HSD rats were similar in pattern of melatonin production to photoresponsive rather than nonphotoresponsive Siberian hamsters [10]. However, HSD rats excreted only about half as much 6-sulfatoxymelatonin per unit body mass as F344 rats, and some individual HSD rats had little or no nocturnal rise in excretion, similar to nonphotoresponsive Siberian hamsters [10].

For this study, a companion study to Price et al. [32], we tested two competing hypotheses. One hypothesis is that differences in photoresponsiveness between F344 and HSD rats are caused by differences in circadian clock function and/or clock outputs cause differences in photoresponsiveness. A competing hypothesis is that differences in photoresponsiveness are caused by differences at the level of melatonin secretion and responses to melatonin [32], rather than by differences in clock function. We tested for circadian differences between young male F344 and young male HSD rats by measuring running wheel activity in short photoperiod, long photoperiod, and constant dark (DD) to assess circadian traits and the degree of nocturnality. Intrinsic tendencies for nocturnality may be most apparent in DD, when individuals must rely entirely on their circadian clock to indicate subjective night and day. Circadian differences could occur in short or long photoperiods as well, though direct effects of light and dark might mask effects of the endogenous circadian rhythm on nocturnality.

## Methods

Under the hypothesis that differences in clock function cause nonphotoresponsiveness in HSD rats, we predicted that HSD rats would be more likely to be arrhythmic or have poorly defined circadian rhythms of activity, because a damaged or altered clock or clock output pathways that result in an inability to track time-of-day or to pass time-of-day information to other areas of the brain would result in poor circadian regulation of activity. For similar reasons, we predicted that HSD rats would have lower nocturnality than F344 rats because HSD rats would assess day and night inaccurately. Low nocturnality in HSD rats might be most extreme in constant dark, when light cues are not available to mask circadian outputs. In addition, if HSD rats were found to be able to entrain activity to the dark period at least in long photoperiod, we predicted a free-running rhythm greater than 24 hours in HSD rats (which results in entrainment even if there is a deficit in the phase delay portion of the phase response curve) along with inability to lengthen the activity period when moved from long days to short days (which might result from inability to properly phase-delay in response to short photoperiod). This prediction follows Puchalski and Lynch [11], who used the nonparametric theory of entrainment [33] in developing a model for nonresponsiveness of Siberian hamsters.

The study followed international standards for animal care and welfare, and was approved by the institutional animal care and use committee at the College of William and Mary (IACUC 0018).

### Experiment 1: Comparison of HSD to F344 male rats in LD

This experiment was designed to compare circadian traits and nocturnality in a photoperiod treatment with light cues that would not normally trigger photoperiodic responses. Activity patterns of young male F344/NHsd rats (breeders from Harlan, Indianapolis, Indiana) and HSD rats (Hsd:Sprague Dawley; breeders from Harlan, Indianapolis, Indiana) were compared in long days (LD), 16 L: 8 D with lights on at 0500 EST (N = 15 F344 rats and 18 HSD rats). Rats were only tested during the four weeks after weaning in order to match the period when F344 rats and other strains are known to show variation in photoperiodic responses [27,28,30,34]. In order to control for potential changes related to age, each individual rat was tested in only one photoperiod treatment.

All rats were gestated and raised in LD (16 L: 8 D with lights on at 0500 EST) prior to weaning at 23 ± 2 days of age. They were then transferred to individual cages with activity wheels (Harvard Apparatus, Holliston, Massachusetts, Rodent Activity Wheel and Cage, Catalog No. 60-1943) and placed in environmental and photoperiod chambers (Revco, Asheville, North Carolina) in groups of up to 12 rats/chamber. Magnetic switches on the running wheels signaled revolutions to an event recorder sending output in 6-minute data collection periods, or 'bins', to a personal computer. Rats were fed a laboratory diet (Harlan Teklad LM – 485 Sterilizable Mouse/Rat Diet 7012, Madison, WI) and tap water ad libitum. Temperature was maintained at 22.5 ± 1°C. Data collection ended after four weeks ± 4 days. The study followed international standards for animal care and welfare, and was approved by the institutional animal care and use committee at the College of William and Mary (IACUC 0018).

### Experiment 2: Comparison of HSD to F344 male rats in SD

This experiment was designed to compare circadian traits and nocturnality in a photoperiod with light cues that would normally trigger reproductive suppression, reduced food intake, and slowed somatic growth in a photoresponsive rat. Activity patterns of young male F344 and HSD rats (N = 16 per group) were compared for short days (SD), 8 L: 16 D with lights on at 0900 EST. Except for the photoperiod treatment, procedures and data collection were as in Experiment 1.

### Experiment 3: Comparison of HSD to F344 male rats in DD

This experiment was designed to compare circadian traits and nocturnality of F344 and HSD rats. Activity patterns of young male F344 rats were compared with those of young male HSD rats (N = 10 per group) in constant darkness (DD). Except for the photoperiod treatment, procedures and data collection were as in Experiment 1.

### Data Analysis

Data collection and analysis of nocturnality and diurnality was carried out using a software package, Tau, generously provided by Roberto Refinetti . Wheel revolutions were recorded in 6-minute bins and plotted as activity records. Preliminary analyses were conducted to test the effect of removing bins with small numbers of wheel rotations. Because variation in nocturnality was most apparent without removing bins, analyses were conducted using all activity. For all statistical analyses, data were taken only from the last 15 days of activity in order to allow rats to adjust to the treatment photoperiod in SD and DD. For rats in LD, data collection was also restricted to the final 15 days of activity in order to match ages across all three experiments. The nocturnality index is defined here as the ratio of time active in the night (or subjective night) to the total time active over 24 hours. Thus, individuals that were more active during the night had a higher nocturnality index. The Tau program also calculated the *tau *(free-running period) by the chi-square periodogram method for each rat in DD. Phase angle of entrainment, activity onset, activity offset, and *alpha *(duration of activity) were calculated following methods slightly modified from Majoy and Heideman [17] and Sullivan and Lynch [35] as described below.

Activity onset was defined as the first bout of running activity lasting at least two 6-min recording bins that was preceded by at least 2 hours with no sustained activity (e.g., activity in no more than one consecutive bin) and followed by additional activity within the next hour. Eye-fitted lines were drawn through these daily activity onsets on an actogram to obtain a mean daily activity onset for each rat. The activity offset was defined as the end of the last bout of activity followed by at least 2 hours of no sustained activity. Again, eye-fitted lines were drawn through the daily offset times on an actogram to obtain a mean offset for each rat. *Alpha *was defined as the difference between activity offset and activity onset. Phase angle of entrainment was defined as the difference between activity onset and lights off (calculated only in SD and LD photoperiods). Unpaired t-tests were performed to test for statistical significance between the two strains of rats in each photoperiod treatment for each circadian parameter.

Mean nocturnality index, *tau*, phase angle of entrainment, activity onset, activity offset, and *alpha *were compared between strains in each photoperiod by unpaired t-tests, with the criteria for statistical significance set at P < 0.05. Because data were taken in multiple runs, run was included as a factor in the analyses. Run did not affect significance tests, and was not considered further.

## Results and discussion

**Figure 1:**
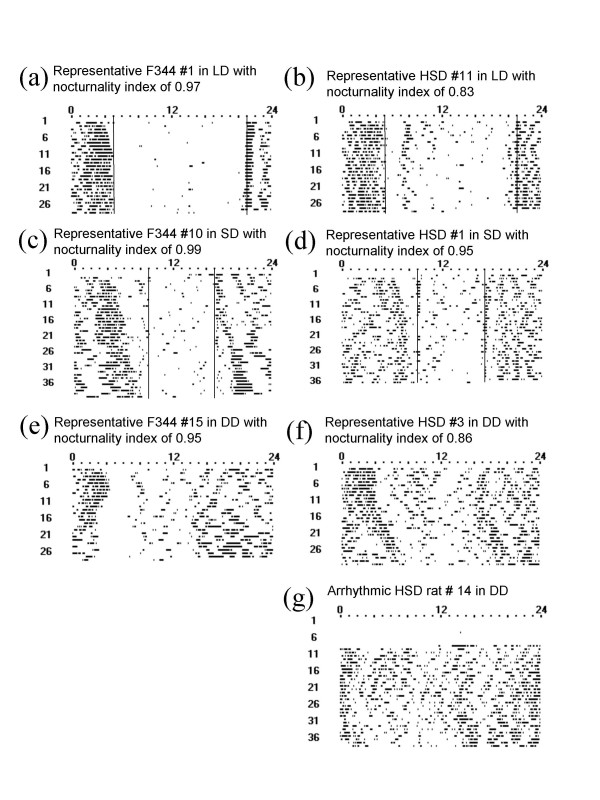
**Representative actograms of F344 and HSD rats.** (a) F344 rat in LD, (b) HSD rat in LD, (c) F344 rat in SD, (d) HSD rat in SD, (e) F344 rat in DD, (f), HSD rat in DD, (g) arrhythmic HSD rat in DD.

**Figure 2:**
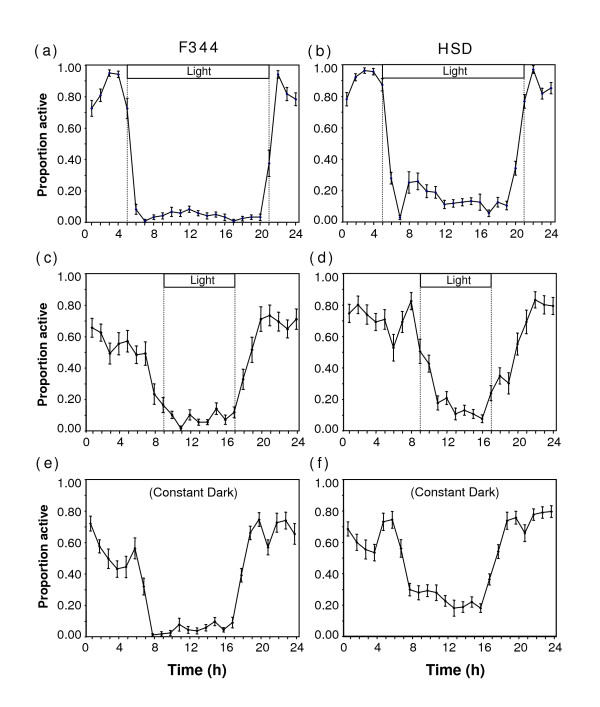
**Proportion of rats active in relation to time of day (Mean +/- SEM).** (a) F344 in LD, (b) HSD in LD, (c) F344 in SD, (d) HSD in SD, (e) F344 in constant dark, and (f) HSD in constant dark. The profiles show the proportion of individual rats active on the running wheel during each hour, averaged over the final four days of activity monitoring. For rats in constant dark, activity was fit to the 24 hour profile by placing the zero time point at the midpoint of activity.

In all three photoperiods, there were strain specific differences in activity patterns. Representative rhythms from individual rats with circadian parameters near the means for their treatment group are shown in Figure 1a–f. Summary data showing the proportion of rats active in each hour are presented in Figure 2.

**Figure 3:**
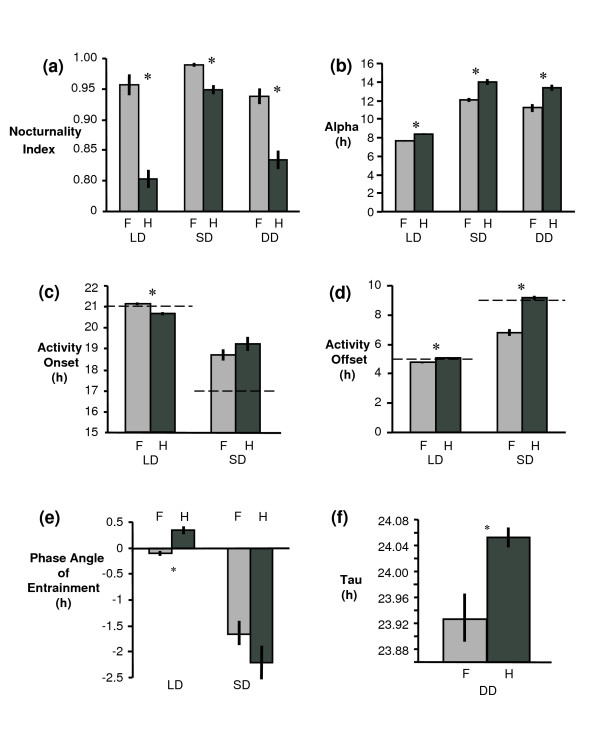
**Circadian parameters for F344 (F) and HSD (H) rats in LD, SD, and DD (Mean +/- SEM).** (a) nocturnality index, (b) *alpha*, the duration of activity, (c) activity onset, with dashed lines indicating the time of lights off, (d) activity offset, with dashed lines indicating the time of lights on, (e) phase angle of entrainment, and (f) *tau*, the free-running rhythm in DD. Asterisks indicate significant differences between F344 and HSD rats.

In LD, SD, and DD, F344 rats had higher nocturnality indices than HSD rats (LD: t = 6.85, p < 0.0001; SD: t = 4.83, p < 0.0001; DD: t = 5.05, p < 0.0001; Figure 3a), with a higher proportion of their activity during the night period than HSD rats (Figure 2). In all three photoperiod treatments, *alpha *was significantly longer in HSD than in F344 rats (LD: t = 5.60, p < 0.0001; SD: t = 4.92, p < 0.0001; DD: t = 3.86, p = 0.0005; Figure 3b). There were differences in activity onset in LD (t = 5.01, p < 0.0001; Figure 3c) but not in SD (t = 1.12, p = 0.27; Figure 3c), and differences in activity offset in both LD (t = 4.28, p = 0.0002; Figure 3d) and SD (t = 8.37, p < 0.0001; Figure 3d). There was a significant difference in phase angle of entrainment in LD (t = 4.95; p < 0.0001; Figure 3e), but not in SD (t = 1.26; p = 0.22; Figure 3e). HSD rats also displayed *tau *longer than 24 h, significantly longer than *tau *of F344 rats (t = 3.01; p = 0.0048; Figure 3f).

Overall, HSD rats tended to extend activity into the light period and also had a higher amount of activity during the light period than F344 rats (Figures 1, 2, &3). Some HSD rats had activity spread so evenly through the constant dark period as to appear nearly arrhythmic (Figure 1g).

Young male HSD and F344 rats differed in multiple circadian parameters of running wheel activity (Figure 3). HSD rats were more likely to begin and end activity during the light period or subjective day in SD, LD, and DD. In addition, HSD rats had more activity during day or subjective day than F344 rats in all three treatments (Figs. 2 &3a). Consistent with Aschoff's prediction for more diurnal animals [36], HSD rats also had *tau *> 24 hours, significantly longer than the < 24 hour *tau *of F344 rats (Figure 3f). However, HSD rats had significantly longer *alpha *than F344 rats (Figure 3b), and both strains entrained well to SD (Figure 2c,d), which is consistent with the hypothesis that differences in melatonin secretion or responses to melatonin cause variation in photoresponsiveness, and inconsistent with the hypothesis that circadian deficits in HSD rats cause nonphotoresponsiveness.

It has been proposed that nonphotoperiodism may occur in Siberian hamsters because they fail to produce an SD pattern of melatonin secretion due to a failure to integrate photoperiodic information by the circadian system [10]. The cause of this failure was associated with a long-duration *tau *(24.04 +/- 0.05), inability to undergo phase delay in response to a light pulse, and a failure to increase *alpha *or entrain properly in SD. While HSD rats have some similarities to nonphotoperiodic Siberian hamsters, the results are not identical. HSD rats have relatively low amplitude 6-sulfatoxymelatonin excretion patterns when adjusted for body mass [32] and a long duration *tau *(24.05 +/- 0.02; Figure 3f), similar to nonphotoperiodic Siberian hamsters [10,11]. However, HSD rats produce 6-sulfatoxymelatonin excretion rhythms that adjust to night length and are similar to those of F344 rats in SD [32], and HSD rats successfully decompress activity to a long-duration pattern when transferred from LD to SD (Figure 3b). We have not tested directly the ability of HSD rats to phase delay, but successful entrainment to SD suggests that HSD rats have the ability to phase delay to achieve entrainment to SD. Thus, while HSD rats show some similarities to nonphotoperiodic Siberian hamsters, the evidence from this study, together with results of Price et al. [32], suggests that the circadian system of HSD rats is able to respond appropriately to photoperiodic information (this study) to cause a short-day melatonin secretion pattern [32], but HSD rats are unable to respond reproductively to a short-day melatonin pattern [32].

In combination with the companion study [32], our results support the hypothesis that differences in how F344 and HSD rats secrete and/or respond to melatonin are responsible for differences in photoresponsiveness. We found significant differences between strains in circadian aspects of wheel running, and these differences indicate that HSD rats tend to have more activity during the day, have a greater tendency toward arrhythmia, and differ in some circadian parameters from F344 rats. However, the differences reported here in circadian parameters and nocturnality do not prevent similar timing of 6-sulfatoxymelatonin excretion rhythms in F344 and HSD, albeit potentially lower in amplitude or even arrhythmic in HSD rats [32]. Rats are normally nocturnal [e.g., [22,24]], and our results suggest that F344 rats have the more species-typical pattern of strong nocturnality, while HSD rats differ from that pattern. Variation in activity and 6-sulfatoxymelatonin excretion has also been reported between other strains of rats [24], but our results suggest there is no necessary relationship between photoresponsiveness and circadian rhythm characteristics in rats. For example, some of the strain variation we report in wheel running activity might be related to restlessness, fearfulness, or intrinsic rewards of wheel running that differ among rat strains.

## Conclusion

In laboratory populations of rodents, circadian variation has been reported to be a cause of variation in responsiveness to photoperiod [10-14]. In contrast, in HSD and F344 rats as well as in wild-source populations of mammals, variation in photoresponsiveness may not be linked to variation in circadian characteristics [16-20]. Uncoupling of the circadian system and seasonal rhythms can be caused experimentally [37] or may occur naturally during part of the year in polar regions [38]. Natural populations may be under strong selection against circadian mutations that have pleiotropic effects on traits such as seasonality. In these natural populations, mutations that act specifically on outputs, such as melatonin sensitivity of the reproductive axis, pelage, body mass or feeding, are more likely to be adaptive or neutral. In contrast, laboratory populations are not under selection to eliminate mutations that alter circadian clock function in ways that affect multiple output systems, including seasonal photoperiodic responses [e.g., [15]], which may explain differences reported previously between wild-source and laboratory populations.

The pattern of differences in photoresponsiveness between HSD and F344 rats, which appear to have little relationship to differences in circadian organization, may indicate greater similarity to wild populations containing natural genetic variation in photoresponsiveness. However, we cannot entirely rule out the possibility that HSD rats are nonphotoresponsive due to differences from F344 rats in circadian organization. Knock-out studies of single circadian clock genes in mice have been reported to cause changes in rhythm parameters, reduced nocturnality or loss of rhythmicity, and also changes in reproductive traits [4]. Nevertheless, it appears more likely that differences between HSD and F344 rats in running wheel activity rhythms, nocturnality, and reproductive photoresponsiveness are merely incidentally correlated. Differences in activity and nocturnality may be caused by subtle differences in circadian organization, while differences in photoresponsiveness are likely due to an independent trait, insensitivity of the reproductive axis, body mass, and food intake to a short-day melatonin pattern [32].

## Competing interests

The authors declare that they have no competing interests.

## Authors' contributions

CDS participated in design of the study, carried out the data collection and preliminary data analyses, and wrote initial drafts of the manuscript. CEJ helped complete data analysis and helped to draft the manuscript. PDH conceived the study, participated in design of the study and data analysis, and developed the final draft of the manuscript. All authors read and approved the final manuscript.
